# Portocaval paraganglioma: A second case report

**DOI:** 10.1016/j.amsu.2022.104236

**Published:** 2022-07-31

**Authors:** Duaa Ghajar, Firas Khana, Mohammed Deeb Zakkor, Hachem AlHussein, Alae Kayyali

**Affiliations:** a Department of Endocrinology Medicine, Aleppo University Hospital (AUH), Aleppo, Syria; b Department of Medical Imaging and Diagnostic Radiology, Aleppo University Hospital (AUH), Aleppo, Syria

**Keywords:** Portocaval paraganglioma, Portal vein, Inferior vena cava, Magnetic resonance spectroscopy, Case report

## Abstract

**Study design:**

Case Report.

**Introduction and importance:**

To report a case of a paraganglioma presenting in an uncommon location in the abdomen.

**Case presentation:**

A 24-year-old man with an abdominal lesion presented with one-year history of severe headaches and palpitations.

**Interventions and outcome:**

The tumor was surgically resected and was later diagnosed as an extra-adrenal paraganglioma.

**Conclusion:**

The unique location of a paraganglioma could prove misleading, making it easier to confuse it with other malignant lesions, but it still can't be excluded, and with proper techniques it can be surgically excised.

## Highlights

• Paragangliomas (PGLs) are slow-growing tumors of neuroendocrine origin.
• PGLs in the portocaval region are considered extremely rare.
• Their unusual location has its own implications that must be carefully considered.
• Clinical findings with imaging techniques and laboratory tests are key to diagnosis.
• Surgical resection remains the gold standard of treatment.

## 1 Introduction

Paragangliomas (PGLs), or glomus tumors, are rare, slow-growing, extra-adrenal tumors of neuro-endocrine origin. They arise from either sympathetic or parasympathetic paraganglia found throughout the body.

While there exist reports of retroperitoneal paragangliomas, a paraganglioma situated between the portal vein and the inferior vena cava has only been described once in the literature.

This paper presents a case of a portocaval paraganglioma mainly for its unique location and the implications it had on patient's management.

This case report has been reported in line with the SCARE criteria [17].

## 2 Case Presentation

A 24-year-old Caucasian man presented with a one-year history of severe headaches not responding to medications, palpitations, sustained hypertension and generalized hyperhidrosis. He also complained of erectile dysfunction and loss of libido.

The patient was non-alcoholic, but he was a light smoker and has smoked less than one pack-year. His past medical history revealed well-controlled diabetes mellitus by metformin and diet. He also had no family or allergy history.

Upon physical examination, the presence of hypertension was confirmed with measurements ranging from 160/90 mmHg and up to 190/100 mmHg.

Differential diagnosis considered the possibility of a primary tumor in the abdomen, including pheochromocytoma (PCC), paraganglioma (PGL) and other adrenal tumors. It even included a possible prolactinoma which was excluded later on due to normal prolactin levels and lack of visual field defects.

Laboratory investigations were carried out. Increased levels of 24-h urine normetanephrine (5933 mcg/dl) were noted. All other tests (CBC, electrolytes, urea, creatinine, TSH, FT4, prolactin, free testosterone and semen analysis) yielded normal results.

Evaluation with ultrasonography of the abdomen showed a hyper-vascular iso-to hypoechoic lesion located in the porta hepatis region, and both the adrenal glands were unremarkable.

Neck ultrasound was normal.

Intravenous (IV) contrast-enhanced computed tomography (CECT) of the abdomen showed a 7.2-cm × 6.5-cm × 5.4-cm, well-defined, irregularly-spherical, heterogenous, arterial enhancing lesion in near proximity of the caudate lobe of the liver, which confirmed the ultrasonography findings.

**Fig. 1:**
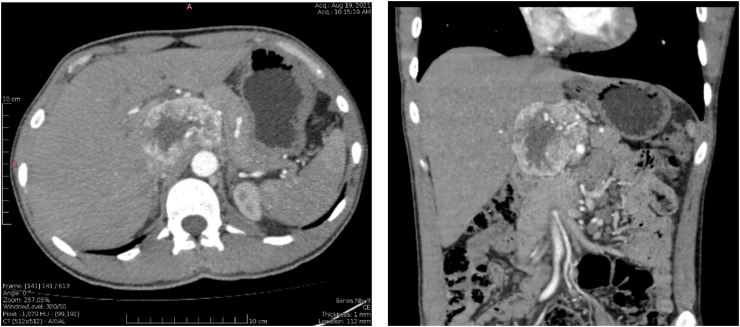
Axial and coronal views in the arterial phase show a heterogenous vividly-enhancing mass between the portal vein and the IVC.

Both axial and coronal views of the mass in the arterial phase of CECT are demonstrated in Fig. 1.

The mass demonstrated no invasion to the caudate lobe, pancreas, portal vein, IVC and the adrenals, as they were all spared, and this was later confirmed by surgery.

While it's true that functional imaging performed with iodine-123 metaiodobenzylguanidine (MIBG) and single photon emission computed tomography (SPECT) has high sensitivity and specificity for detecting primary tumors and metastases, in our case it didn't help as the lesion showed no uptake of the isotope.

Further evaluation with gadolinium-enhanced magnetic resonance imaging (GEMRI) was performed, and showed the lesion to be iso-to hypo-intense on T1-weighted images, and iso-to hyper-intense on T2-weighted and fat saturation images, with intense and heterogenous enhancement with no signs of invasion.

**Fig. 2:**
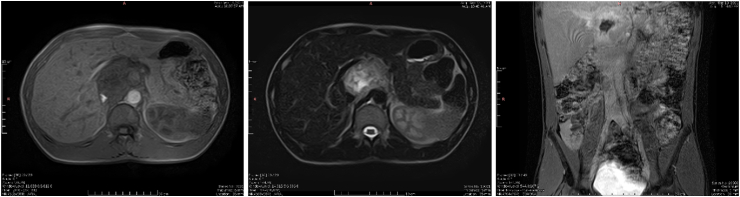
Axial and coronal planes demonstrating an iso-to hypo-intense mass on unenhanced T1-weighted images (left), and iso-to hyper-intense on fat-saturated T2-weighted images (center), with avid and heterogenous enhancement (hyperintense rim with hypointense central region) on gadolinium-enhanced T1-weighted images (right).

Both axial and coronal views of the lesion on GEMRI are shown in Fig. 2.

No enlarged lymph nodes were seen.

**Fig. 3:**
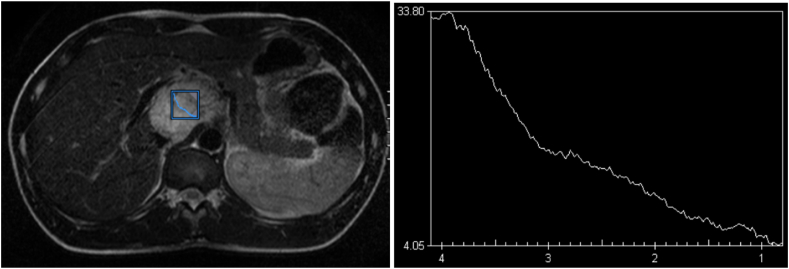
Axial plane demonstrating VOI adaptation prior to spectroscopy (left), and the resulting ^1^H-MRS spectra with no evidence of a succinate peak at 2.42 ppm albeit poor water suppression (right).

Additional sequences for in vivo proton magnetic resonance spectroscopy (^1^H-MRS) were performed, and resulting spectra, which were acquired using single-voxel spectroscopy (SVS) along with manual shimming, showed no evidence of a singlet succinate peak at 2.42 ppm as seen in Fig. 3.

**Fig. 4:**
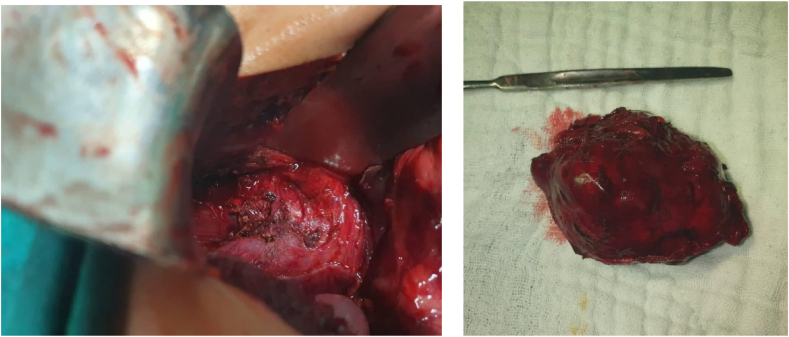
Intraoperative (left) and postoperative (right) pictures of the portocaval paraganglioma.

The patient was initially planned for laparoscopic surgery but was later converted to open surgery due to size and location of the tumor, and the nature of its surroundings. The precise anatomical location of the tumor was identified, anterior to the inferior vena cava, with the portal vein situated anteromedially and the pancreas inferiorly (Fig. 4). No invasion into surrounding structures was seen.

Histopathological findings confirmed the tumor to be an extra-adrenal paraganglioma.

Following surgery, the patient's progression was uneventful, and lab investigations were normal at follow-up visits 3- to 6-month after.

## 3 Discussion

We report a rare case of a 24-year-old man with prolonged complaints of headaches, palpitations and hypertension, whose biochemical tests revealed elevated normetanephrine levels in 24-h urine.

Abdominal imaging showed a well-defined mass extending between the portal vein and inferior vena cava, it was surgically removed and the presence of a functional paraganglioma was further confirmed by histopathology.

We have two possible explanations as to why the iodine-123 metaiodobenzylguanidine (MIBG) single photon emission computed tomography (SPECT) study gave a false-negative result, the most logical being the patient undergoing a contrast-enhanced computed tomography (CECT) study 2-weeks prior, which is highly plausible.

The other explanation is that some paragangliomas associated with SDHB gene mutations might give false-negative results thanks to their rapidly growing nature. Though, this is highly unlikely as such tumors are mostly metastatic and suffer from poor prognosis, which isn't the case here as the imaging findings clearly show, and as later confirmed by surgery. This fact is furthermore supported by the lack of a singlet succinate peak on ^1^H-MRS.

Postoperative surveillance was carried out for 6 months, with 24-h urine normetanephrine levels being normal when measured at the second- and fourth-week intervals following surgery. Hypertension had been normalized and antihypertensive agents were discontinued. Patient was recommended for normetanephrine level measurement on a quarterly basis during the first year following surgery, then semiannually for at least 5 years, and was also recommended for weekly blood pressure monitoring for the first year and monthly thereafter.

## 4 Conclusion

Functional paragangliomas in the portocaval region are an extremely rare presentation of paragangliomas. The unusual location makes it easier to confuse them with other abdominal malignant lesions, particularly retroperitoneal ones, but the diagnosis of a PGL still can't be excluded. Utilizing proper radiographic and imaging techniques is key to diagnosis, and total surgical resection remains the gold standard treatment for managing such tumors.

## Sources of funding

This research did not receive any specific grant from funding agencies in the public, commercial, or not-for-profit sectors.

## Ethical approval

This case report did not require review by the ethics committee in Aleppo University Hospital (AUH), Aleppo, Syria.

## Registration of research studies

Not applicable.

## Consent

Written informed consent was obtained from the patient for publication of this case report and accompanying images. A copy of the written consent is available for review by the Editor-in-Chief of this journal.

## Author contribution

• Duaa Ghajar: contributed in data collection and interpretation, and writing the paper (First Author)
• Firas Khana: contributed in data collection and interpretation, study design and writing the paper (Coauthor #1 and Corresponding Author)
• Mohammed Deeb Zakkor: contributed in data interpretation, study concept and writing the paper (Coauthor #2)
• HACHEM AlHussein: contributed in reviewing the paper (Coauthor #3)
• Alae Kayyali: contributed in reviewing the paper (Coauthor #4)

## Guarantor

Firas Khana.

## Provenance and peer review

Not commissioned; externally peer reviewed.

## Declaration of competing interest

All authors declared no conflict of interest.
